# Meeting abstracts from the Annual Conference “Clinical Genetics of Cancer 2022”

**DOI:** 10.1186/s13053-023-00253-5

**Published:** 2023-07-12

Edited by Jan Lubiński

The Supplement Editor declares no competing interests

Funding: "Clinical Genetics of Cancer 2022" was subsidised by the Polish Ministry of Education and Science within the framework of the programme "Excellent Science (Doskonała Nauka)" (project no. DNK/SP/550146/20221).

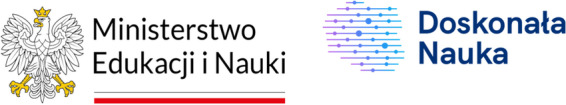



## A1 Desmoid tumours, familial adenomatous polyposis and sporadic infiltrative fibromatosis

### Rodney J. Scott^1^, Raewyn Billings^2^, Kathy Tucker^3^

#### ^1^Discipline of Medical Genetics, School of Biomedical Sciences and Pharmacy, University of Newcastle, Callaghan, NSW, 2308, Australia; ^2^BC Cancer Agency, Department of Molecular Oncology, Vancouver, V5Z 1L3, Canada; ^3^Hereditary Cancer Clinic, The Prince of Wales Hospital, Randwick, NSW, 2031, Australia

*Hereditary Cancer in Clinical Practice 2023,* **21(1):**A1

From time to time young adult patients will present with a desmoid tumour, which arouses suspicion of Familial Adenomatous Polyposis (FAP) and the risk of colorectal cancer. Desmoid tumours are unequivocally associated with FAP and many studies have revealed that there is a generalised genotype/phenotype correlation with the likelihood of a FAP patient presenting with such a tumour. In addition, the entity familial infiltrating fibromatosis is considered allelic to FAP since pathogenic variants in *APC* have been identified that are associated with this disorder. Thus patients presenting with desmoid disease should be screened for pathogenic variants in *APC* to either exclude FAP or include it. The majority of patients, however, do not carry a germline pathogenic variant, but rather a somatic mutation in *CTNNB1* (*β-catenin*). Currently, there are only four mutations in *CTNNB1* that have been associated with sporadic desmoid disease, c.121A>G; c.122C>T; c.133T>C and c.134C>T; with the majority of mutations occurring at position c.121A>G. The current literature to date suggests that “sporadic” desmoid tumour patients are a result of mutations within *CTNNB1* and that inherited pathogenic variants in this gene are not compatible with foetal development. At present, the dogma indicates that only *CTNNB1* is associated with sporadic desmoid disease and that *APC* is uniquely associated with inherited forms of disease. Notwithstanding, there remain a percentage to sporadic desmoid tumour patients who do not have a genetic diagnosis. Given that there are FAP families that do not display an overt FAP phenotype but to present with multiple cases of desmoid disease, we reasoned that some of the sporadic desmoid tumour patients who did present with a *CTNNB1* mutation may be the result of an *APC* mutation located within a region of *APC* correlated with desmoid tumour development.

## A2 Understanding of germline genetic basis of familial cancer

### Kari Hemminki^1,2^

#### ^1^Biomedical Center, Faculty of Medicine and Biomedical Center in Pilsen, Charles University in Prague, 30605 Pilsen, Czech Republic; ^2^Division of Cancer Epidemiology, German Cancer Research Center (DKFZ), Im Neuenheimer Feld 580, D-69120, Heidelberg, Germany

*Hereditary Cancer in Clinical Practice 2023,* **21(1):**A2

Thanks to panel sequencing and genome-wide sequencing methods, large amounts of germline sequencing data have recently become available. We sought to compare these results with population-based family history data. Family studies are able to describe aggregation of any defined cancers in families. The Swedish Family-Cancer Database is the largest of its kind in the world, covering the Swedish families through nearly a century with all cancers in family members since the start of national cancer registration in 1958. The database allows estimation of familial risks, ages of cancer onset and the proportion of familial cancer in different family constellations. Here, we review the proportion of familial cancer for all common cancers and specify them based on the number of affected individuals. With the exception of a few cancers, age of onset of familial cancer is not different from all cancers combined. The highest proportions of familial cancer were found for prostate (26.4%), breast (17.5%) and colorectal (15.7%) cancers, but the proportions of high-risk families with multiple affected individuals were only 2.8%, 1% and 0.9%, respectively. A large sequencing study on female breast cancer found that *BRCA1* and *BRCA2* mutations could account for 2% of the cases (subtracting the proportions in healthy individuals) and that all germline mutations accounted for 5.6% of the cases. Early age of onset was a distinct feature of only *BRCA* mutations. In heritable colorectal cancer, Lynch syndrome genes dominate. Large studies on penetrance in Lynch syndrome have shown an approximately linear increase in risk from 40-50 years up to age 80 years. Interesting novel data revealed a strong modification of familial risk by unknown factors. High-risk germline genetics of prostate cancer is characterized by *BRCA* and other DNA repair genes. *HOXB13* encodes a transcription factor which contributes to germline risk of prostate cancer. A strong interaction was shown with a polymorphism in the *CIP2A* gene. The emerging germline landscape of common cancers can be reasonably accommodated by family data on these cancers as to high-risk proportions and age of onset.

## A3 Genetic predisposition to colorectal cancer: Identification of novel cancer susceptibility genes

### Försti Asta

#### Hopp Children's Cancer Center (KiTZ), Heidelberg, Germany; Division of Pediatric Neurooncology, German Cancer Research Center (DKFZ), German Cancer Consortium (DKTK), Heidelberg, Germany

*Hereditary Cancer in Clinical Practice 2023,* **21(1):**A3

About 15% of colorectal cancer (CRC) patients have first-degree relatives affected by the same malignancy. However, for most families the cause of familial aggregation of CRC is unknown. To identify novel high-to-moderate-penetrance germline variants underlying CRC susceptibility, we performed whole exome (WES) and whole genome sequencing (WGS) in Polish CRC families showing a Mendelian inheritance pattern. After WES or GWS, we used our in-house developed Familial Cancer Variant Prioritization Pipeline to identify novel cancer predisposition variants. We identified both nonsense, missense and 5’UTR variants involved in the regulation of innate immune response (*SLC15A4*), apoptosis and AKT pathway (*PTK7*), reactive oxygen species and mucus biology (*CYBA*, *TRPM4*), Wnt signaling (*APCDD1*) and histone modification (*HDAC5*) and in a protooncogene (*SRC*). Some of the identified variants may show they effect according to a synergistic or polygenic model. Our findings contribute to the identification of unrecognized genetic causes of familial CRC.

## A4 Mesalamine for colorectal cancer prevention program in Lynch syndrome

### Ann-Sofie Backman

#### Karolinska Universitetssjukhuset Gastrocentrum - Stockholm, Sweden Karolinska Universitetssjukhuset Gastrocentrum - Stockholm, Sweden

*Hereditary Cancer in Clinical Practice 2023,* **21(1):**A4

This is a multicenter, multinational, randomized, 2-arm, double-blind, phase II clinical study with 2000mg mesalamine (5-ASA) or placebo in LS patients for a 2-year treatment. 260 tumor free carriers of a known genetic mutation in a major MMR gene (including patients in which the polyps are endoscopically removed) will be randomized 1:1 to receive 2000mg mesalamine or placebo. Patients will be identified through local or national registries and through collaboration with sites. Tumor free patients, assessed by white light high resolution colonoscopy, will be randomized to the study. Blood and stool samples will be collected for analysis of microbiota, ctDNA and potential biomarkers. Biopsies of the normal tissue of ascending colon and rectum will be taken at the first and the last colonoscopy.

The aim of the study is to investigate the effect of regular treatment with mesalamine (5-ASA) on the occurrence of any colorectal neoplasia, tumor multiplicity (the number of detected adenomas/carcinomas) and tumor progression in LS patients.

Tumor multiplicity and tumor progression (severity of the neoplastic lesions) will be investigated.

## A5 Screening and efficacy of sporadic and hereditary CRC detection in low incidence population

### Elsako Pavel

#### State Research Institute, Innovative Medicine Center, Vilnius, Lithuania; Public Institution Centro Poliklinika, Vilnius, Lithuania

*Hereditary Cancer in Clinical Practice 2023,* **21(1):**A5

The patients survival in case of early detection of the cancer by FOBT screening and colonoscopy is an indicator of incidence and mortality from CRC. However mortality rates are high in countries with relatively low incidence such as in Moldova, Russia, Montenegro, Poland and Lithuania. For intense in Lithuania the CRC age-standardized incidence rate is 23.4 and mortality rates 13.7 while in the Netherland comparative rates are 40.2 and 13.4 respectively. The first time during 2000 year in Lithuania was introduced in medicine practice guaiac Haemoccult test for early diagnosis of CRC improving and to investigate the possible development of CRC and diagnoses in patients after once testing. Deadline of follow-up for possible development cancer and diagnosis in 374 patients [256 (68%) female and 118 male (32%)] were once tested by Haemoccult was 2008 September (median 8, 7 years). The risk of cancer development and diagnosis was calculated by standard incidence ratio (SIR) use statistic program PY2. In the 243 (66.9%) patents including with positive CRC family history 48(18.8%) female and 22 (18.6%) male, who were tested and did not obtained positive test, SIR for CRC was 1, 09; in the 120 (33.1%) patients, who have obtained positive test, SIR was 2.04 for CRC and 3.07 for all cancer. Any one a CRC did not diagnosed in stage-I (TNM). The results of this study suggest that early CRC detection by once testing Haemoccult is impossible. In 2009 Lithuania started a national Program for CRC screening by fecal immunochemical testing (FIT) and colonoscopy. The screening program was implemented in June 2009 using the FIT (OC-Sensor test, Eiken,Tokyo Japan) with automated reading techniques in one of the biggest Vilnius city Centro Polyclinic. The target population according to criteria in age 50-74 years for potential screening was 45 330 subjects: female 27 909(61.6%) and 17 421(38.4%) male. Patients whose samples revealed an FIT value Hb>100 –ng/ml of buffer underwent colonoscopy. The rate participation we calculated every 2 years because the patents after initial testing with negative FIT test necessary performed regular every 2 year repeating test. Overall 35 689 – 13 904(39%) male and 21 785 (61%) female subject from a potential target population were accepted for screening. The participation rate for least once screening over the 7 years (every 2 years) was 78.7%. The participation rate in screening calculated every 2 years 1-4 round was 33.6%, 35.1%, 402%, 23.7% respectively for female and male. Estimated that from 35 689 participated in screening patients in 176(0.98%) was diagnosed CRC cases and from 9 641 non-participated patients was diagnosed 94(0.98%) CRC cases. After calculation of comparative efficacy for diagnosis of CRC by stage I-IV and ten years survival of screened and non-screened patients were not significantly different P=0.128908 and P=0.3898 respectively.

Screening of average risk population is limited because the criteria of age are 50-74 years and potential young (below 50 years) subjects with high risk to hereditary cancer including Lynch Syndrome are not accepted in screening. In Lithuania in same period with population screening in 2009 was introduced in medical practice a screen first colon cancer tumour by MSI and IHC staining according to histological criteria and young age below 50 years. We study to estimate the efficacy of cancer diagnoses in young patients below 50 years who had tumour failed to express MLH1, MSH2 and MSH6 use staining MSI and IHC. For period 2009-2019 were diagnosed 54 suspected for Lynch syndrome patients (32 man and 22 women). Any one a CRC did not diagnosed in stage-I (TNM). The distribution of diagnosed CRC stage in each stage were: Stage I-0, II- 19(35.2%) III- 21(38.8%), IV-10(18.5%), unknown-4(7.5%).

Early CRC detection by once testing is impossible. The participation rate for at least once screening over the 7 years of the study was 78.7% with participation rate in each round (1-4) was less than EU guideline set minimum 45% and did not improve a detection of CRC by stage P=0.13 and ten years survival of screened and non-screened of CRC patients is not significantly different P=0.39. Farther research is needed to help to determinate and stratify various risks, such as LS for the development of CRC in low incidence population.

## A6 Recommendation for preventive adnexectomy in MLH1 and MSH2 mutation carriers

### Kuświk Magdalena^1^, Gromowski Tomasz^2^, Dębniak Tadeusz^1^, Kurzawski Grzegorz^1^, Cybulski Cezary^1^, Gronwald Jacek^1^, Huzarski Tomasz^1^, Lubiński Jan^1^

#### ^1^Department of Genetics and Pathology, Pomeranian Medical University, Szczecin, Poland; ^2^Human Genome Variation Research Group & Genomics Centre, Malopolska Centre of Biotechnology, Jagiellonian University, Cracow, Poland

*Hereditary Cancer in Clinical Practice 2023,* **21(1):**A6

Lynch syndrome (LS) is a common hereditary cancer predisposition syndrome. It is caused by mutation in one of four DNA mismatch repair (MMR) genes: *MLH1*, *MSH2*, *MSH6*, *PMS2* or deletion of last exons in *EPCAM* gene. Mutation carriers are at high risk of developing colorectal cancer (CRC) and endometrial cancer (EC). They have also increased risk of some extra-colonic cancers such as ovarian, small bowel, urinary, biliary tract, gastric, and brain tumors. Ovarian cancer (OC) is third most commmon cancer in Polish LS women constitutes about 5% of all LS cancer. There is no consensus guidelines for OC prevention in LS women.

The aim of this study was to evaluate and compare ovarian cancer risk in our series of 289 families with identified pathogenic mutations (referred as Lynch syndrome families) to the general population.

We observed increased risk of OC in Polish LS families in copmarision to the general polulation. Moreover, high risk of ovarian cancer was found for LS women under 50 years of age. Furthermore 6 out of 17 (35%) early-onset patients from LS families died from ovarian cancer within 2 years of diagnosis.

Due to the increased risk of ovarian cancer in LS women and low efficiency of the gynecological screening, for female carriers of a pathogenic variant in *MLH1* and *MSH2* genes prophylactic adnexectomy should be recommended after 35year of age. Additionally because of the small number of cases of pathogenic mutations in the *MSH6*, *PMS2* and *EPCAM* gene, the validity of prophylactic adnexectomy cannot be demonstrated.

## A7 The spectrum of mutations in BRCA1/2 detected by NGS in pancreatic cancer patients considered for treatment with PARP inhibitor

### Tyka Magdalena^1^, Hińcza- Nowak Kinga^1^, Szczepaniak Magdalena^1^, Miłek-Krupa Justyna^1^, Siołek Monika^2^, Kopczyński Janusz^3^, Góźdź Stanisław^4^, Kowalik Artur^1^

#### ^1^Department of Molecular Diagnostics, Holy Cross Cancer Center, Kielce, Poland; ^2^Genetic Clinic, Holy Cross Cancer Center, Kielce, Poland; ^3^Department of Surgical Pathology, Holy Cross Cancer Center, Kielce, Poland; ^4^Clinical Oncology, Holy Cross Cancer Center, Kielce, Poland

*Hereditary Cancer in Clinical Practice 2023,* **21(1):**A7

**Introduction**

Mutations in the BRCA1/2 genes are responsible for hereditary breast cancer, ovarian cancer, prostate cancer as well as pancreatic cancer. The results of pancreatic cancer treatment are still unsatisfactory with a five-year survival rate of only 8.8% (KRN) in Poland (11% in the US). Recently, PARP inhibitors have been introduced as a promising treatment option for pancreatic cancer patients with mutations in the BRCA1/2 genes.

**Aim**

The purpose of this study is to review the spectrum of detected mutations in BRCA1/2 genes in patients referred for molecular diagnosis due to consideration of PARPi treatment.

**Material and Methods**

Between 2020 and 2022, 143 patients with stage III and IV pancreatic cancer were tested for BRCA1/2 genes by NGS in DNA isolated from 116 (82%) blood samples and 28 (18%) cancer tissue samples.

**Results**

In 3/28 (11%) tissue samples showed DNA degradation and no test result was obtained. No mutations in BRCA1/2 were detected in any of the tissue samples tested. All 8 pathogenic mutations were detected in blood samples. Two mutations were detected in the BRCA1 gene and six in BRCA2. In total, mutations were detected in 5.7% (8/140) of the patients studied.

**Conclusions**

BRCA1/2 diagnosis for pancreatic cancer should be carried out by NGS because the mutations are mostly confined to the BRCA2 gene and additionally scattered throughout the gene. In one case, founder mutations were detected in the BRCA1 gene.

## A8 Simple method of detection of the most common PALB2 and RECQL genes mutations

### Andrzej Pławski

#### Institute of Human Genetics, Polish Academy of Sciences, Poznan, Poland

*Hereditary Cancer in Clinical Practice 2023,* **21(1):**A8

Breast cancer is the most common cancer in women and in majority it is thought to be sporadic. 10-15% of breast cancer cases is considered to be hereditary. The most common genetic susceptibility is caused by mutation in BRCA1 and BRACA2 gene mutations which are responsible for 5-10 % of all familial cases. Cybulski et al. and Erkko et al. described the mutation in PALB2 and RECQL genes mutations increasing risk of breast cancer. In Europe the recurrent mutation of this gene are c.1667_1667+3delAGTA in the RECQL gene and c.509_510delGA and c.172_175delTTGT in the PALB2 gene. In our region also frequent is deletion of exon 9 of the PALB2. We developed the method for fast and easy identification of those 4 mutation. The method is based on C-HRM technique developed by our team few years ago which let to detect the point mutations and copy number variations in the same time. The Patent Office of the Republic of Poland granted the patent (official number P.436028) to the Institute of Human Genetics PAS for the invention entitled: "Method and diagnostic kit for detecting RECQL and PALB2 gene variants in Polish patients with breast cancer". Authors of the invention are: Andrzej Pławski, Jan Lubiński, Cezary Cybulski, Zdenek Kleibl, Emilia Lis-Tanaś, Paweł Boruń.

## A9 Characterization of liquid-biopsy based microRNA profiles of *BRCA1/BRCA2* positive patients – a preliminary study

### Tokarski Miron^1,2^, Małodobra-Mazur Małgorzata^1,2^, Cierzniak Aneta^2^, Kilar Ewa^3^

#### ^1^Department of Molecular Techniques, Faculty of Medicine, Wroclaw Medical University, Wrocław, Poland; ^2^Genomtec SA, Wrocław, Poland; ^3^Department of Clinical Pharmacology, Medical University of Wroclaw, Wroclaw Poland

*Hereditary Cancer in Clinical Practice 2023,* **21(1):**A9

MicroRNAs (miRNAs or miRs) are small non-coding RNAs that play a crucial role in both cell development and differentiation, by influencing post-transcriptional regulation of several protein-coding genes. In the past, it was thought that miRNAs could only limit the expression of target genes by interacting with the mRNA transcript. However, more recent studies have shown that miRNAs may also be responsible for a post-transcriptional increase in gene expression. The final effect of miRNA's influence on mRNA and the resulting increase in expression seems to be dependent on specific cell conditions, mRNA sequence and other cofactors. There are numerous mechanisms responsible for the miRNA-dependent reduction of gene expression, which include both translation initiation as well as post-initiation mechanisms. In both types of interactions, miRNPs play a key role in reducing gene expression. The literature suggests that there are several microRNAs (miRNAs, miRs) that exhibit either increase or decrease of expression in liquid-biopsy samples during early onset of several malignancies including breast and ovarian cancer. It is important to mention that scientific literature suggests ethnic-related changes in the expression of microRNA molecules, which requires tailoring of the assays to local requirements. By selecting proper miR molecules, it is possible to develop highly specific and sensitive test for cancer detection, prognosis or even selecting tailored therapy.

In this presentation we are demonstrating preliminary results covering four patients assessed in two different timepoints over the span of monitoring and therapy period as a part of National Prevention Programme of BRACA1/BRACA2 positive patients.

Testing was performed on freshly collected serum samples that undergo microRNA extraction process and expression assessment utilising gold standard for miR detection – Real-Time RT-PCR with TaqMan® Array Card: TaqMan™ Advanced miRNA Human Serum/Plasma Cards able to detect 188 distinctive microRNAs.

Obtained results are promising, exhibiting the ability to differentiate expression profiles for two different timepoints when compared to healthy individuals. This might enable differentiation between early onset of disease and progression in the future. In order to fully evaluate the preliminary results, in the next step we plan to evaluate additional samples of BRCA1/BRCA2 positive patients both not exhibiting symptoms of malignancies as well as on different stages of active cancer therapy out of over 140 currently banked with array screening together with additional healthy patients which will allow exclusion of both individual and environmental factors.

## A10 PTEN-HTS – Genotype-phenotype correlations - a single institution’s experience

### Nowakowska Dorota^1^, Kwiatkowska Ewa^2^, Kotlarek Marta^3^, Kolanowska Monika^3^, Wójcicka Anna^3^, Janiec – Jankowska Aneta^2^, Tysarowski Andrzej^2^

#### ^1^Genetic Counselling Unit, Cancer Prevention Department, The Maria Sklodowska - Curie National Research Institute of Oncology, Warsaw, Poland; ^2^Department of Genetic and Molecular Diagnostics of Cancer The Maria Sklodowska - Curie National Research Institute of Oncology, Warsaw, Poland; ^3^Warsaw Genomics, Warsaw, Poland

*Hereditary Cancer in Clinical Practice 2023,* **21(1):**A10

**Introduction**

*PTEN* hamartoma tumor syndrome (PTEN-HTS or PHTS, OMIM 158350, ORPHA:306498) is the collective term for a group of syndromes, including Cowden syndrome, adult Lhermitte-Duclos disease, Bannayan-Riley-Ruvalcaba syndrome, and Proteus -like syndrome, caused by germline mutations in the *PTEN* gene, located on 10q23.3. Inheritance of PHTS is autosomal dominant. The syndrome is rare, with an incidence of 1 in 200000, however it is very likely to be underestimated. Hamartomas are a common manifestation of the PHTS. The lifetime risk (LTR) of developing breast cancer for women is about 77- 85%, for endometrial cancer 19 -28%. LTR for thyroid cancer for the carriers is approximately 21-38%, for renal cell cancer 15- 34%, for colorectal cancer 9%-16%, for melanoma up to 6%. Affected individuals usually have macrocephaly and frequently present with rare benign mucocutaneous lesions. Diagnostic criteria for PHTS are regularly updated by the National Comprehensive Cancer Network.

**Material and methods**

In our Institution NGS sequencing has been made available for clinical use since 2018 year. Our multigene panel includes *PTEN* gene. During the last 4 years at our Genetic Counselling Unit we identified 6 unrelated patients with symptoms prompting PHTS diagnosis. The probability of finding *PTEN* mutation was calculated for each individual using The Cleveland Clinic Adult Clinical Scoring System.

All the patients were evaluated by the same clinical geneticist before and after the test. Written informed consent for the genetic testing had been obtained by the same medical doctor. After establishing diagnosis all the patients received detailed genetic counsel and were included into surveillance programme, according to the guidelines (ERN-Genturis/ NCCN -combined).

DNA was extracted from the peripheral blood leukocytes using QIASymphony QIAGEN technique. For the first group we used Illumina Platform NextSeq500, for the last 3 patients we used Ion AmpliSeq On-Demand DNA Panel by Thermo Fisher.

The pathogenicity of the variants/ mutations was assessed according to the Recommendations from the ClinGen PTEN Expert Panel and checked in ClinVar database.

**Results**

PTEN – HTS (PHTS) diagnosis has been confirmed in 6 patients (4 women, 2 men) carrying 6 different germline pathogenic *PTEN* mutations: one in the 1^st^ exon, one in exon 3/9, two different in exon 5/9, one in exon 6/9, one in intron 7, one also intronic - after exon 8. All the patients had OFC ≥ 60 cm. Both men presented with benign only tumours – mainly skin lesions and gastrointestinal polyps and both of them had established diagnosis of autism spectrum disorder. Two of 4 women presented with multiple cancer ( breast, endometrial, ovarian in one and breast and rectal cancer in the second one). Unexpected diagnoses in our group of patients were: thyroid cancer at the age of 5 years, schwannoma and neurofibroma.

**Conclusions**

This is the first Polish study of the genotype – phenotype correlations among patients with PHTS. We were not able to prove any founder/ recurrent mutations in this group. We found 6 different pathogenic mutations in our unrelated 6 patients. Our study confirms value of the established clinical criteria and online tools for identifying PTEN-HTS/Cowden syndrome in Polish patients. The routine measuring of head circumference in hereditary cancer clinics seems to be a good recommendation.

## A11 Increasing significance of methylation biomarkers in clinical cancer management

### Wojdacz Tomasz K

#### Independent Clinical Epigenetics Laboratory, Pomeranian Medical University, Szczecin, Poland

*Hereditary Cancer in Clinical Practice 2023,* **21(1):**A11

In general terms epigenetic mechanisms of gene expression regulation alter gene expression without changing primary gene sequence. There is a number of epigenetic mechanisms that orchestrate gene expression and render cell phenotype. Malfunctioning of those mechanisms leads to disease such as neoplastic transformation. In principle each of those mechanisms can be targeted by treatment or a become source of biomarkers. However, currently only assays targeting DNA methylation changes are finding significantly increasing use in clinical disease management. DNA methylation is a covalent addition of the methyl groups to cytosines within CpG dinucleotide and methylation of the promoter sequences interferes with gene transcription.

There is already substantial evidence that biomarker assays targeting disease related methylation changes can effectively be utilized as biomarkers at all stages of the clinical disease management: from risk assessment through early diagnosis and treatment personalization to post treatment surveillance. Specifically, one of the largest clinical trials have recently shown that methylation biomarker-based cancer detection in liquid biopsy allows to detect cancer in patients with remarkable specificity and sensitivity. Moreover, detectable in blood methylation of *BRCA1* gene has been proposed as biomarker of predisposition to breast and ovarian cancers. The methylation of *MGMT* gene has long been used to guide treatment of glioblastoma multiforme patients. And recently, the genome wide profiling methylation has been suggested as alternative to standard histopathology method for the classification of brain tumours by the World Health Organization.

With those developments methylation biomarkers can significantly contribute and change cancer management as well as play a significant part in personalized medicine. In my talk I will review current applications of the methylation biomarkers in clinical disease management.

## A12 Constitutional *BRCA1* promoter methylation and risk of ovarian cancer

### Ogrodniczak Alicja^1^, Jaroch Angelika^1^, Prajezendanc Karolina^1^, Białkowska Katarzyna^1^, Menkiszak Janusz^2^, Huzarski Tomasz^1,8^, Gronwald Jacek^1^, Dębniak Tadeusz^1^, Cybulski Cezary^1^, Tomiczek-Szwiec Joanna^3^, Szwiec Marek^4^, Jasiówka Marek^5^, Blecharz Paweł^6^, Kluz Tomasz^7^, Stawicka-Niełacna Małgorzata^8^, Mądry Radosław^9^, Tołoczko-Grabarek Aleksandra^1^, Byrski Tomasz^10^, Lubiński Jan^1^, Jakubowska Anna^1,11^

#### ^1^Department of Genetics and Pathology, Pomeranian Medical University, Szczecin, Poland; ^2^Department of Gynecological Surgery and Gynecological Oncology of Adults and Adolescents, Pomeranian Medical University, Szczecin, Poland; ^3^Department of Histology, Department of Biology and Genetics, Faculty of Medicine, University of Opole, Opole, Poland; ^4^Department of Surgery and Oncology , University of Zielona Góra, Zielona Góra, Poland; ^5^Department of Chemotherapy Ludwik Rydygier Memorial Specialist Hospital, Zlotej Jesieni 1, Krakow, Poland; ^6^Department of Gynaecologic Oncology, Maria Sklodowska-Curie National Research Institute of Oncology, Krakow Branch, Krakow, Poland; ^7^Department of Gynecology and Obstetrics, Institute of Medical, Sciences, Medical College of Rzeszow University, Rzeszow, Poland; ^8^Department of Clinical Genetics and Pathology, University of Zielona Góra, Zielona Góra, Poland; ^9^Chair of Oncology, Department of Gynecological Oncology, Poznan University of Medical Science, Poznań, Poland; ^10^Department of Oncology and Chemotherapy, Pomeranian Medical University, Szczecin, Poland; ^11^Independent Laboratory of Molecular Biology and Genetic Diagnostics, Pomeranian Medical University, Szczecin, Poland

*Hereditary Cancer in Clinical Practice 2023,* **21(1):**A12

Epigenetic mechanisms, including DNA methylation, play an important role in carcinogenesis. DNA methylation in promoter of a gene region can lead to inactivation of this gene by transcriptional silencing processes. Constitutional *BRCA1* promoter methylation has been shown to potentially correlate with the risk of ovarian cancer.

The aim of this study was to evaluate the association of *BRCA1* promoter methylation detected in blood-derived DNA with the risk of ovarian cancer.

The study group consisted of 649 unselected ovarian cancer patients and 795 healthy controls. All women were negative for 13 *BRCA1/2* germline mutations common in Polish population. Methylation status of *BRCA1/2* gene in ovarian cancer patients was assessed using methylation-sensitive high-resolution melting (MS-HRM), and classified as positive or negative. Statistical analysis was done using Fisher exact test.

We found that *BRCA1* promoter methylation was present in 10.3% of unselected ovarian cancer cases and in 6.5% of healthy controls, what corresponded to slightly (about 50%) increased risk of ovarian cancer (OR 1.65, 1.12-2.40, p = 0.01).

The results suggest that *BRCA1* promoter methylation can have a potential role in ovarian cancer susceptibility, but larger more in-depth studies are required.

## A13 Role of trace elements in early detection of cancer

### Pietrzak Sandra^1^, Lener Marcin^1^, Marciniak Wojciech^2^, Baszuk Piotr^1^, Derkacz Róża^2^, Lubiński Jakub^3^, Jaworowska Ewa^3^, Wójcik Janusz^4^, Kładny Józef^5^, Jakubowska Anna^1^, Cybulski Cezary^1^, Gronwald Jacek^1^, Dębniak Tadeusz^1^, Huzarski Tomasz^1^, Scott Rodney J^6^, Narod Steven A^7^, Lubiński Jan^1,2^

#### ^1^Department of Genetics and Pathology, International Hereditary Cancer Center, Pomeranian Medical University, Szczecin, Poland; ^2^Read-Gene, Grzepnica, Poland; ^3^Clinic of Otolaryngology and Laryngological Oncology, Pomeranian Medical University, Szczecin, Poland; ^4^Department of Thoracic Surgery and Transplantation, Pomeranian Medical University, Szczecin, Poland; ^5^Department of General and Oncological Surgery, Pomeranian Medical University, Poland; ^6^Medical Genetics, Hunter Medical Research Institute, Priority Research Centre for Cancer Research, Innovation and Translation, School of Biomedical Sciences and Pharmacy, Faculty of Health and Medicine, University of Newcastle, Pathology North, John Hunter Hospital, Newcastle, Australia; ^7^Women's College Research Institute, Dalla Lana School of Public Health, University of Toronto, Toronto, Canada

*Hereditary Cancer in Clinical Practice 2023,* **21(1):**A13

**Introduction**

According to the International Agency for Research on Cancer (IARC) 19 million new cases of cancer are diagnosed every year worldwide. In Poland above 171 000 new cases of cancer are diagnosed each year. Due to a large number of cancers, it is important to try to identify new early detection markers. For many years, elements’ effects on human life and health, including the occurrence of cancer, have been widely studied.

In this research we want to find whether selected elements (Se, Cu, Cd) levels in serum/blood could be related to the cancer occurrence.

**Methods**

For analysis, 10 mL of peripheral blood was collected for elements levels in serum/blood and were determined at the time of diagnosis of cancer, before the start of treatment. Elements levels were quantified by inductively coupled mass spectrometry (ICP-MS). We assigned patients to one of four categories of elements levels based on the distribution of selected element levels in the entire study group. All statistical calculations were performed using: R: A language and environment for statistical computing. R Foundation for Statistical Computing, Vienna, Austria (R version 4.04). The studies was conducted in accordance with the Helsinki Declaration and with the consent of the Ethics Committee of Pomeranian Medical University under the number KB-0012/73/10. All participants provided written consent to be enrolled in the herein studies.

**Findings**

Low selenium level was associated with an odds ratio of 5.81 (95% CI: 2.18 to 16.52; p = 0.0001) for lung cancer, 5.44 (95% CI: 2.14 to 14.62; p < 0.001) for laryngeal cancer and 13.78 (95% CI 6.31 to 29.82; p < 0.001) for colorectal cancer. Moreover, odds ratio for colorectal cancer for those in the highest quartile of copper level (versus the lowest) was 12.7 (95% CI: 4.98 to 32.3; p < 0.001). In research on blood cadmium level as a marker for early lung cancer detection - odds ratio for those in the highest quartile of cadmium level (versus lowest) was four-fold higher (OR = 4.41, 95% CI: 2.01 to 9.67; p < 0.01). The association was present in former smokers (OR = 16.8, 95% CI: 3.96 to 71.2, p < 0.01), but not in current smokers (OR = 1.23, 95 % CI: 0.34 to 4.38) or in never smokers (OR not defined).

**Conclusion**

Low selenium level (<60 μg/l; serum) may be a marker for early detection of the lung, larynx and colorectal cancers. High copper level (>930 μg/l; blood) may be a marker for early detection of colorectal cancer. High level of cadmium (>0.47 μg/l; blood) may be a marker for early detection of lung cancer, especially in former smokers. It seems that determining the level of Se, Cd and Cu could be a marker for selection for control examinations in surveillance as a valuable complement to existing diagnostic procedures.

## A14 Role of trace elements in cancer prevention

### Białkowska Katarzyna^1^, Marciniak Wojciech^2^, Derkacz Róża^2^, Baszuk Piotr^1,2^, Pietrzak Sandra^1^, Lener Marcin^1^, Ogrodniczak Alicja^1^, Prajzendanc Karolina^1^, Huzarski Tomasz^1^, Gronwald Jacek^1^, Oszurek Oleg^1^, Cybulski Cezary^1^, Dębniak Tadeusz^1^, Jakubowska Anna^1,3^, Lubiński Jan^1,2^

#### ^1^Department of Genetics and Pathology, International Hereditary Cancer Center, Pomeranian Medical University, Szczecin, Poland; ^2^ Read – Gene SA, Grzepnica, Poland; ^3^Independent Laboratory of Molecular Biology and Genetic Diagnostics, Pomeranian Medical University, Szczecin, Poland

*Hereditary Cancer in Clinical Practice 2023,* **21(1):**A14

Since the data from prospective studies are available, one of the critical challenges in oncology is to reverse carcinogenic effect of elements.

One of major concerns in Polish women population is arsenic. Marciniak et. al. shown that arsenic is one of risk factors for breast cancer for carriers and non-carrier of deleterious variants in BRCA1 gene. Most of arsenic is ingested with food. Therefore the diet became the first trial of blood arsenic lowering study. After 30 months of low arsenic diet, 55% of participants showed blood arsenic decreased by 55%.

Selenium has antagonistic effect on arsenic, both in absorption and metabolism. We have conducted an analysis of selenium to arsenic ratio. There is an optimal range of Se/As ratio which is correlated with significantly lower cancer incidence.

Some papers suggest also that vitamin C may be beneficial in lowering blood arsenic levels. We have performed a pilot study with orally administered vitamin C supplement. After 30 days of daily intake of 1g of vit C, arsenic level was significantly decreased in 95% participants.

In men, blood lead level is associated with 7 times increased risk of cancer. In a pilot study we found a substance that is capable to reduce blood lead level to background level.

In conclusion, blood arsenic, selenium and lead are strong cancer risk factors. It seems that we are able to reverse the adverse effect of these element by simple diet modification or vitamin C supplementation.

## A15 Elements in cancer treatment

### Lubinski Jan^1,2^; Marciniak Wojciech^2^; Derkacz Róża^2^; Gronwald Jacek^1^; Cybulski Cezary^1^; Huzarski Tomasz.^1^; Dębniak Tadeusz^1^; Lubiński Jakub^3^; Lener Marcin^1^; Jakubowska Anna.^1^; Szwiec Marek^4^; Pietrzak Sandra^1^; Baszuk Piotr^1^; Rogoża-Janiszewska Emilia^1^; Białkowska Katarzyna^1^; Rudnicka Helena^1^; Rowińska Karolina^1^; Falco Michał^5^; Wójcik Janusz.^6^; Grodzki Tomasz^5^; Sun Ping^7^; Scott J Rodney^8^; Narod Steven^7,9^

#### ^1^Department of Genetics and Pathology, International Hereditary Cancer Center, Pomeranian Medical University, Szczecin, Poland; ^2^Read-Gene, Grzepnica, Poland; ^3^Clinic of Otolaryngology and Laryngological Oncology, Pomeranian Medical University, Szczecin, Poland; ^4^Department of Surgery and Oncology, University of Zielona Góra, Zielona Góra, Poland; ^5^Regional Oncology Centre, Szczecin, Poland; ^6^Department of Thoracic Surgery and Transplantation, Pomeranian Medical University, Szczecin, Poland; ^7^Women's College Research Institute, Toronto, Canada; ^8^Medical Genetics, Hunter Medical Research Institute, Priority Research Centre for Cancer Research, Innovation and Translation, School of Biomedical Sciences and Pharmacy, Faculty of Health and Medicine, University of Newcastle; Pathology North, John Hunter Hospital, Newcastle, Australia; ^9^Dalla Lana School of Public Health, University of Toronto, Toronto, Canada

*Hereditary Cancer in Clinical Practice 2023,* **21(1):**A15

During the last few years we performed series of studies on correlation between serum levels of panel of elements – As, Se, Zn, Cu, Mn, Cd and Pb, and survival in malignant tumours of various sites: cancers of the breast, prostate, lung, larynx, pancreas and malignant melanoma. Except of clinically advanced cancers of the lung and pancreas (stages II-IV) in all other malignancies there were found unequivocal (statistically significant or strong tendencies) correlations between serum Se, Zn, and Cu levels and patients 5 and/or 10 years survivals. Interestingly, optimal levels of elements were similar in different tumours: Se - ~100μg/l, Zn - ~1100 μg/l and Cu - ~900μg/l.

## A16 SELINA – all cause mortality decreased by selenium blood level optimization

### Lubinski Jan^1,2^, Marciniak Wojciech^2^, Derkacz Róża^2^, Huzarski Tomasz^1,2,3^, Gronwald Jacek^1,2^, Cybulski Cezary^1,2^, Dębniak Tadeusz^1^, Ashuryk Oleg^1^, Tołoczko-Grabarek Aleksandra^1^, Stachowska Ewa^4^, Lubinski Wojciech^5^, Godlewski Dariusz^6^, Tomczak Anna^6^, Posmyk Renata^7^, Kilar Ewa^8^, Czajka D^6^, Dach Krzysztof^9^, Janowska Magdalena^10^, Jasiewicz Andrzej^10^, Kozak-Klonowska Beata^11^, Kluz Tomasz^10^, Kluza Katarzyna^10^, Leśniewicz R^7^, Siołek Monika^11^, Szwiec Marek^12^, Tomiczek-Szwiec Joanna^12^, Wiśniowski Rafał^13^

#### ^1^International Hereditary Cancer Center, Department of Genetics and Pathology, Pomeranian Medical University, Szczecin, Poland; ^2^Read-Gene SA, Grzepnica, Poland; ^3^Department of Clinical Genetics and Pathology, University of Zielona Góra, Zielona Góra, Poland; ^4^Department of Human Nutrition and Metabolomics, Pomeranian Medical University, Szczecin, Poland; ^5^II Department of Ophthalmology, Pomeranian Medical University, Szczecin, Poland; ^6^Center of Cancer Prevention and Epidemiology OPEN, Poznań, Poland; ^7^Podlasie Medical Center "Genetics", Genetic Clinic, Bialystok, Poland; ^8^Independent Public Health Care Center, Oncology Clinic, "Kite" Hospital, Świdnica, Poland; ^9^Voivodship Hospital in Łomża, Department of Pathology and Oncological Prevention, Łomża, Poland; ^10^Podkarpackie Oncology Center, Rzeszów, Poland; ^11^Holly Cross Cancer Center, Kielce, Poland; ^12^Opole Oncology Center, Opole, Poland; ^13^Beskid Oncology Center, Bielsko-Biała, Poland

*Hereditary Cancer in Clinical Practice 2023,* **21(1):**A16

Acknowledgements: Putresza Ewa, Gibaszek Renata

Grant: INNOMED/I/16/NCBR/2014

SELINA is a clinical study on influence of selenium blood levels optimization on cancer occurrence and all cause mortality in females from families with increased risk of hereditary breast cancers.

7456 women at the age of at least 40 years have been recruited from centers in Poland. According to planned protocol, 40 months after recruitment, decoding for safety reason was performed. Compliance rate was 81%. Intervention did not cause undesired effects in any arm.

By contrast, in some sub-groups very promissive correlations have been found. The strongest positive effects were noted in subgroups of BRCA1(-) (without occurrence of Polish founder constitutional mutations): Selenium deficiency (blood level <98μg/l) on supplement Sel-BRCA1® (ethanol solution of sodium selenite), placebo or diet and selenium excess (blood level >108μg/l) on diet. In all above subgroups combined, all cause mortality was decreased more than 3 times in females who achieved blood Selenium level 98-108 μg/l 5 and 1162 vs 35 and 2509; p=0.0154, OR=3.2, CI = 1.3-8.3. The greatest effect was achieved in subgroup supplemented with Sel-BRCA1®: 1 and 346 vs 12 and 396; p=0.0044, OR=10.5, CI=1.4-81.1. The risk of cancers was decreased provided Arsenic levels were added to analyses. The index risk was created of Se level (μg/l) plus As level multiplied by 50.

In subgroup of females under the age of 50, risk index 125-145 was correlated with more than 5 times decreased risk of cancers: 2 and 610 vs 21 and 966; p=0.0021, OR=6.6, CI=1.5-28.4.

## A17 Report on the implementation of Early Detection and Prevention Programs for Cancers in Families with High, Hereditary Risk of Disease - Module I and Module II - in the group of patients covered by diagnostics and genetic counselling

### Gordon-Sönmez Zofia^1^, Kałużewski Tadeusz^2,3^, Stempień Marek^4^, Kubiak Izabela^2,5^, Kucharska Dorota^2,5^, Bednarek Michał^2^, Sałamunia Jordan^2^, Kępczyński Łukasz^3^, Kubicki Tobiasz^6^, Trzciński Radzisław^4,7^, Bartosińska – Dyc Anna^1^, Gach Agnieszka^3^, Szwalski Jarosław^8^, Kałużewski Bogdan^2,5^

#### ^1^Gynecology and Obstetrics Unit, Zduńska Wola County Hospital, Zduńska Wola, Polska; ^2^ Laboratory of Medical Genetics GENOS, Łódź, Polska; ^3^Department of Genetics, Polish Mother's Memorial Hospital Research Institute, Łódź, Polska; ^4^ Department of General and Colorectal Surgery with Subdivision of Oncological Surgery, John Paul II Regional Hospital in Bełchatów, Bełchatów, Polska; ^5^ Regional Cancer Prevention Centre, Zduńska Wola County Hospital, Zduńska Wola, Polska; ^6^Sokrates Medical Center, Sieradz, Polska; ^7^ Institute of Medical Sciences, Collegium Medium of Jan Kochanowski University in Kielce, Kielce, Polska; ^8^Laboratory Cytopath, Łódź, Polska

*Hereditary Cancer in Clinical Practice 2023,* **21(1):**A17

Within 46 months, the Clinics and Laboratories of Medical Genetics "Genos" and the Regional Cancer Prevention Center at the Hospital in Zduńska Wola implemented Module I and Module II of the National Cancer Control Program coordinated by the Ministry of Health, providing genetic counseling and conducting genetic diagnostics in people with high risk of cancer development. The research covered 1024 people, identifying 129 cases with germline mutations. The conducted research allowed to establish individualized prophylactic and therapeutic procedures in mutation carriers. In many cases, the implementation of the Program turned out to be important for families with a high hereditary risk of developing the disease. Continuation and expansion of the Program seems to be crucial for achieving the goals of personalized medicine.

## A18 Selected organizational aspects of care for high-risk families according new program NFZ (National Health Found) in Poland in 2022

### Prusaczyk Artur^1,2,3^, Gronwald Jacek^4,1^, Płochocka Halina^1^, Kalisz Małgorzata^1^, Kordowska Anna^1^, Michałowska Jolanta^1^, Karczmarz Sabina^1^, Wielogórska-Jastrzębska Anna^1,5^, Lubiński Jan^4^, Bidziński Mariusz^2^, Bogdan Magdalena^6,1^, Piątek Szymon^2^

#### ^1^Medical and Diagnostic Center in Siedlce, Siedlce, Poland; ^2^ National Institute of Oncology in Warsaw, Warsaw, Poland; ^3^Lazarski University in Warsaw, Warsaw, Poland; ^4^ Pomeranian Medical University in Szczecin, Szczecin, Poland; ^5^Mazovian Regional Hospital in Siedlce, Siedlce, Poland; 6 Medical University of Warsaw, Warsaw, Poland

The program of care for families with a high, hereditary cancer risk - breast cancer, ovarian cancer, colorectal cancer, endometrial cancer, in Poland in the years 2008-August 2022 was based on specific funding from the Ministry of Health under National Program for Combating Cancer. The program has been a significant success. However, he encountered development barriers, in particular in terms of the increase in the population covered by care and in terms of coordinating cooperation with primary health care and some oncology centers.

From September 2022, in accordance with the NSO implementation plan, the National Health Fund became the financing organization the program. The principles of organizing and financing the components of the program were partially changed, adapting them to the general principles of creating health products by the National Health Fund and their valuation by national a health technology assessment and pricing agency-the AOTMiT.

Each of the services financed by the National Health Fund covers two stages: 1- Counseling and genetic tests, 2- Supervision and diagnostic tests.

In the Polish low-integrated health care system, there are island solutions or there are no formal institutions or mechanisms responsible for obtaining population results for the defined health contribution. Implementation takes place through the spontaneous involvement of interested medics or medical organizations and entities.

The material discusses proposals for a smooth and effective transition and improvement of care for the population under care. Solutions have been proposed that allow for higher population propagation, using existing resources by coordinating care and integrating cooperation with primary health care, outpatient care and gynecology, oncological surgery and clinical oncology departments and areas.

Efforts should focus on:

1. education of medical, non-medical and managerial and coordination staff in the field of new competences,
2. building care plans and organizing e-consultations
3. intensification of systemic cooperation with the National Health Fund-(NFZ) and E-Health Center-(CEZ), mainly through IT integration using the P1 platform and through the use of integrated office and hospital peripheral systems and systematic modification of the components of the National Health Fund's contracts.
4. using the implementation of care coordination in Primary Care (POZ) and creation of an electronic national cancer registry- (eKRN) to build new effective cooperation in the field of oncological prevention programs, especially in the field of surveying.

Proper implementation of care for high-risk families according new program NFZ in the population may make it possible to achieve an improvement in the results of treatment of the above-mentioned cancers as well as other cancers in Poland in the coming years.

## A19 The genetic risk of thyroid cancer among breast cancer patients

### Cieszyńska Monika^1^, Kluźniak Wojciech^1^, Wokołorczyk Dominika^1^, Cybulski Cezary^1^, Huzarski Tomasz^1,2^, Gronwald Jacek^1^, Falco Michał^3^, Dębniak Tadeusz^1^, Jakubowska Anna ^1, 4^, Derkacz Róża^1, 5^, Marciniak Wojciech^1,5^, Lener Marcin^1^, Woronko Karolina^1^, Mocarz Dominika^1^, Baszuk Piotr^1^, Bryśkiewicz Marta^1^, Narod Steven A^6^, Lubiński Jan^1,5^

#### ^1^International Hereditary Cancer Center, Department of Genetics and Pathology, Pomeranian Medical University, Szczecin, Poland; ^2^Department of Clinical Genetics and Pathology, University of Zielona Góra, Zielona Góra, Poland; ^3^West Pomeranian Oncology Center, Radiation Oncology Department, Szczecin, Poland; ^4^Independent Laboratory of Molecular Biology and Genetic Diagnostics, Pomeranian Medical University, Szczecin, Poland; ^5^Read-Gene S.A., Grzepnica, Poland; ^6^Women's College Research Institute, Women's College Hospital, Toronto, Canada

*Hereditary Cancer in Clinical Practice 2023,* **21(1):**A19

**Introduction**

Coincidence of breast cancer with thyroid cancer has been demonstrated in literature previously. It has been recognized that breast cancer survivors have almost twofold increased risk of developing thyroid cancer and the risk is higher than the risk of breast cancer in thyroid cancer survivors. Several factors have been indicated to be involved in both thyroid and breast cancer development. Two genes were identified to be connected with coexisting breast and thyroid cancer: CHEK2 and PARP4. To our knowledge, no study has examined the risk of developing metachronous thyroid cancer in breast cancer patients with mutation in CHEK2 or other genes.

**The aim of study**

The aim of this study is to identify the genes of high risk of thyroid cancer among breast cancer patients.

**Patients**

We recruited 10 869 breast cancer patients. The available information included the age of breast cancer diagnosis, deaths, year of breast cancer surgery, the treatment oncology center, lymph node status, tumor size, histopathology, estrogen-receptor status, progesterone-receptor status, HER2 status, multicentricity, bilaterality, previous treatment (chemotherapy, radiotherapy, hormonotherapy), adnexectomy, other concomitant cancers in probands and family history. Two cohorts were selected: 6755 women with breast cancer diagnosed at age below 51 and 4114 women with breast cancer diagnosed at age above 50. Among 10 869 breast cancer patients, 93 patients (0,9%) developed thyroid cancer in probands. 93 patients with both breast and thyroid cancer were called “research group” and 10 776 patients with breast cancer without thyroid cancer were called “control group”.

**Methods**

The DNA of peripheral blood lymphocytes of patients was examined for the presence of selected germline mutations in two phases. First one was focused on identification of hereditary mutations (including founder mutations) in BRCA1, BRCA2, CHEK2, NBN, RECQL, NOD2, PALB2 and CDKN2A using PCR methods. Then, DNA of 51 patients were examined using NGS (next-generation sequencing) to recognize mutation in 19 selected genes: APC, ATM, BRCA1, BRCA2, CDH1, CDKN2A, CHEK2, MLH1, MUTYH, MSH2, MSH6, NBS1, PALB2, PTEN, PMS2, RAD51C, RAD51D, STK11 and TP53.

**Results**

In the group of patients with breast cancer diagnosed at age below 51 and above 50, the results of BRCA1/2, NOD2, CDKN2A, NBN, PALB2 and RECQL mutation were not statistically significant. The frequency of CHEK2 mutation was studied separately. In the group of 6755 patients with breast cancer diagnosed at age below 51, CHEK2 mutations were present in 10 of 52 (19,2%) breast-thyroid cancer patients and in 585 of 6703 (8,7%) breast cancer patients (OR 2.49; P = 0.01). Missense mutations were seen in 7 of 52 (13,5%) patients from research group and in 417 of 6703 (6,2%) controls (OR 2.34; P = 0.04). Similar tendency was found in the group of protein-truncating mutations (5,8% vs 2,6%), but the results were not statistically significant. In the group of 4114 patients with breast cancer diagnosed at age above 50, CHEK2 mutations were present in 8 of 41 (19,5%) breast-thyroid cancer patients and in 324 of 4073 (8,0%) breast cancer patients (OR 2.81; P = 0.01). The missense mutations were seen in 6 of 41 (14,6%) patients from the research group and in 247 of 4073 (6,1%) controls (OR 2.66; P = 0.03). 2 protein-truncating mutations were detected in research group and 79 mutations in control group (OR 2.59; P = 0.19). Among 51 breast-thyroid cancer patients, 15 mutations were detected using NGS method including 3 new pathogenic or likely pathogenic mutations in genes - TP53 (c.1024C>T), ATM (c.6095G>A) and PTEN (c.852dupA). Among breast cancer patients, 53 thyroid cancers (0,49%) were diagnosed after breast cancer diagnosis (SIR 4.4). In the group of 919 CHEK2 mutation carriers, 10 patients developed thyroid cancer (SIR 9.6). Median time to thyroid cancer diagnosis after breast cancer diagnosis was 5 years. Among 104 breast cancer patients with thyroid cancer in relatives, the frequency of CHEK2 mutation was higher than in the group of breast cancer patients without thyroid cancer in probands or relatives (11,5% vs 8,5%). Breast cancers in women with coexisting thyroid cancer and CHEK2 mutation had tendency to express different histopathological and clinical features – tumors had more often positive estrogen and progesterone receptor status (especially in the group of breast cancer diagnosed below 51 years), were more often multifocal, none was bilateral nor had HER2 overexpression.

**Conclusions**

1. Breast cancer patients have 1,5-fold increased risk of thyroid cancer, especially during 6 years after breast cancer diagnosis (SIR 4.4).
2. Breast cancer patients with CHEK2 mutations have 2-3-fold increased risk of thyroid cancer. The risk is independent on the age of breast cancer diagnosis, type of CHEK2 mutation and is the highest during 5 years after breast cancer diagnosis (SIR 9.6).
3. Next-generation sequencing (NGS) enable to detect new germline mutations among breast-thyroid cancer patients.
4. Screening for thyroid cancer should be considered among breast cancer patients with CHEK2 mutation.

